# Distal radial artery as an alternative approach to forearm radial artery for perioperative blood pressure monitoring: a randomized, controlled, noninferiority trial

**DOI:** 10.1186/s12871-022-01609-5

**Published:** 2022-03-09

**Authors:** Jingwei Xiong, Kangli Hui, Miaomiao Xu, Jiejie Zhou, Jie Zhang, Manlin Duan

**Affiliations:** 1 Department of Anesthesiology, Jinling Hospital, Jinling School of Clinical Medicine, Nanjing Medical University, Nanjing, Jiangsu 210002 People’s Republic of China; 2 Department of Anesthesiology, Affiliated Jinling Hospital, Medical School of Nanjing University, Nanjing, Jiangsu 210002 People’s Republic of China

**Keywords:** Distal radial artery, Arterial catheterization, Blood pressure monitoring, Noninferiority

## Abstract

**Background:**

The novel distal radial artery (dRA) approach is a popular arterial access route for interventional cardiology and neurointerventions. We explored the dRA as an alternative site to the classic forearm radial artery (RA) for perioperative blood pressure monitoring. We hypothesized that dRA catheterization is noninferior to RA for the first attempt success rate.

**Methods:**

This was a single-center, prospective, randomized controlled, noninferiority study. Adult patients who underwent elective surgery at the Jinling Hospital from May 2021 to August 2021 were enrolled. The primary endpoint was to test the noninferiority of the first attempt success rate between the groups. Secondary endpoints included anatomical characteristics, catheterization time, arterial posterior wall puncture rate, postoperative compression time, dampened arterial pressure waveforms, and complications.

**Results:**

Totally, 161 patients who received either dRA (*n* = 81) or RA (*n* = 80) catheterization were analyzed. The first attempt success rates were 87.7 and 91.3% in the dRA and RA groups, respectively, with a mean difference of − 3.6% (95% CI, − 13.1 to 5.9%). The dRA diameter and cross-sectional area were significantly smaller than those of the RA (*P* < 0.001). The subcutaneous depth of dRA was significantly greater than that of the RA (*P* < 0.001). The dRA had a longer catheterization time (*P* = 0.008) but a shorter postoperative compression time (*P* < 0.001). The arterial posterior wall puncture rate of dRA was significantly higher than that of the RA (*P* = 0.006). The dRA had fewer dampened arterial waveforms than RA (*P* = 0.030) perioperatively.

**Conclusions:**

The dRA is a rational alternative approach to RA for perioperative arterial pressure monitoring and provides a noninferior first attempt success rate.

**Trial registration:**

This study is registered in the Chinese Clinical Trials Registry (registration number: ChiCTR2100043714, registration date: 27/02/2021).

## Background

Invasive arterial catheterization in patients who undergo surgery is routinely used for monitoring the hemodynamic status, frequent arterial blood gas sampling, and in the absence of noninvasive blood pressure monitoring [1].

The forearm radial artery (RA) is a recommended site to perform catheterization due to its superficial location, collateral blood supply with the ulnar artery, and limited complications [2, 3]. It is also used in a variety of medical procedures, such as flap transplantation [4], hemodialysis arteriovenous fistula creation [5] and bypass grafting [6]. However, the arterial waveforms are often troubled by wrist flexion [7]. When taking arterial blood pressure reading, wrist flexion often causes artifact waveforms; hence, a different puncture approach is required in some cases.

The distal radial artery (dRA) was first described by Amato et al. [8] in 1977. It is the distal part of the radial artery located at the anatomical snuffbox, which is a triangular depression region consisting of extensor pollicis longus, extensor pollicis brevis, and extensor retinaculum. The scaphoid and trapezium bones form the floor of this region [9]. In recent years, dRA has been popularly catheterized for cardiac and neurosurgical interventions [10–12]. However, there are limited data regarding dRA catheterization for invasive blood pressure monitoring.

Therefore, we performed a trial to explore dRA at the anatomical snuffbox as a replacement for RA in the field of hemodynamic monitoring. Considering the result of pretest, we hypothesized that dRA provides a noninferior first attempt success rate in arterial catheterization, within bounds of the preset noninferiority margin of − 15%.

## Methods

This single center, prospective, randomized controlled, noninferiority study received approval from the Ethics Committee of Jinling Hospital (approval number: 2021NZKY–013-02). Patient recruitment was conducted after registration in the Chinese Clinical Trials Registry (registration number: ChiCTR2100043714, registration date: 27/02/2021). The protocol of the study was performed in accordance with the Declaration of Helsinki.

### Study participants

Patients scheduled for elective surgery in our hospital between May 2021 and August 2021 were enrolled. Each patient signed informed consent. Eligibility criteria included American Society of Anesthesiologists (ASA) Physical Status I to III patients aged 18 to 80 years. Exclusion criteria were the presence of impalpable artery, communication disorder, puncture site infection, peripheral vascular disease, blood coagulation disorder, negative modified Allen test or body mass index (BMI) > 30 kg·m^− 2^.

### Randomization

Study participants were randomly allocated to the dRA or RA group (1:1 ratio) using computer-generated sequences (Excel, Microsoft, USA). An independent investigator (K. H.) placed randomization sequences into numbered sealed envelopes, which were opened in the presence of the participants after obtaining written consent. This study was an open-label design.

### Study treatments

After admission to the operating room, all patients were applied to routine monitoring, which included electrocardiograph (ECG), heart rate (HR), and oxygen saturation (SpO_2_). Patients were placed supine, awake, and the puncture side arms were extended on padded arm boards (dRA group, forearm pronated with the anatomic snuffbox facing upward [10]; RA group, forearm supinated with wrist dorsiflexion up to 30° [13]). We used an ultrasound unit (Wisonic, China) with a 4-15 MHz linear probe. The image depth was set at 25 mm, and the gain settings were adjusted optimally. The contralateral arm was used for patients with previous catheterization of one radial artery.

Anatomic landmarks were used to identify the arterial locations: the anatomical snuffbox and styloid process of the radius for dRA and RA, respectively. To reduce observer bias, we performed each measurement thrice by three independent investigators (J. X., M. X., and J. Z.) on either short or long axis view. The average value was used for statistical analysis.

We palpated the strongest pulsation site around the anatomic landmark, and positioned the ultrasound probe on it. The arterial image in the short-axis plane was captured and saved. Afterwards, the probe was rotated anticlockwise by 90°, and the arterial image in the long-axis plane was saved. To minimize measuring errors, we adjusted probe strength so as not to compress the artery.

**Fig. 1:**
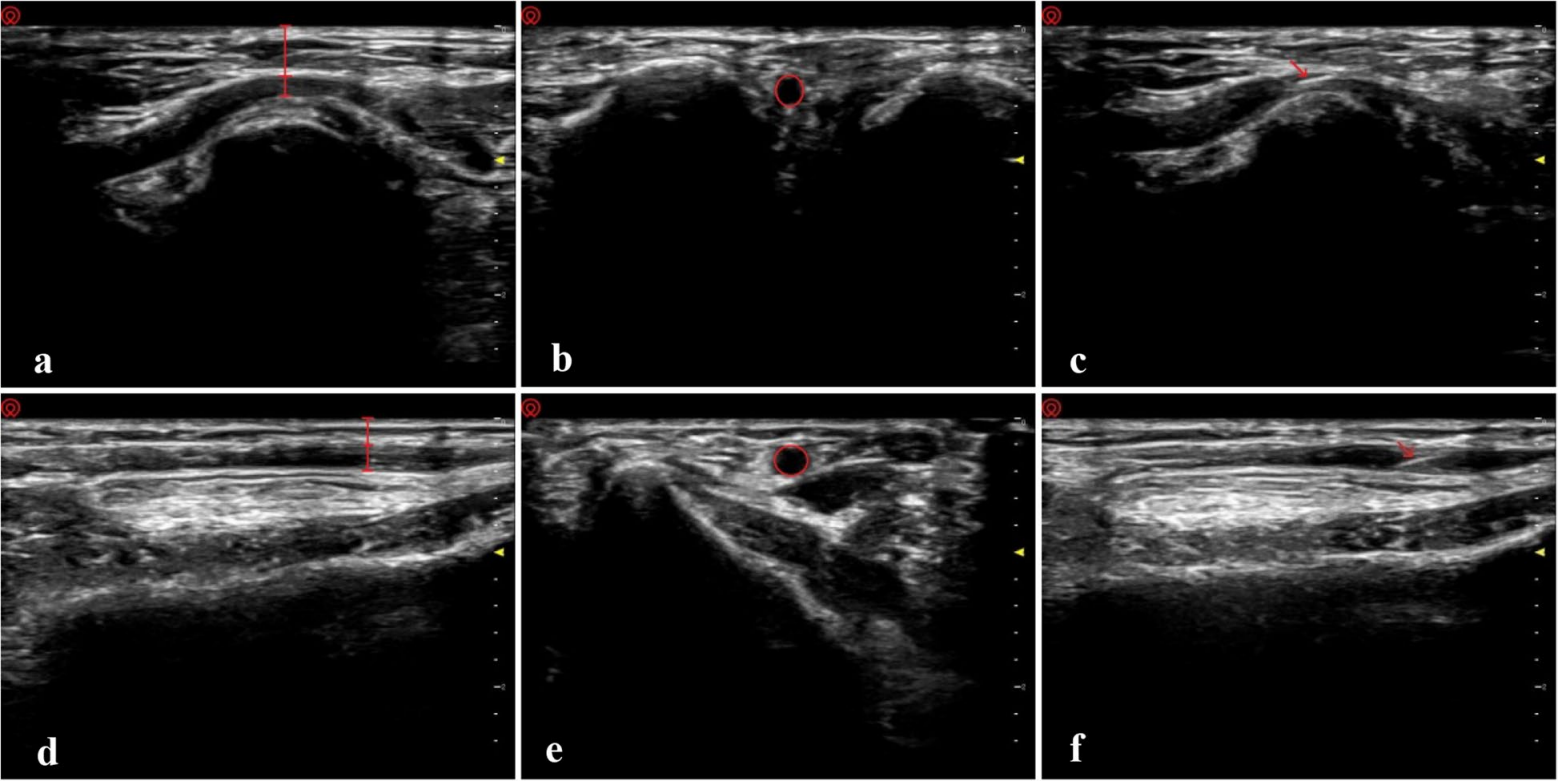
Ultrasound measurements and ultrasound-guided arterial catheterization. **a**, **b**, **c**, distal radial artery (dRA); **d**, **e**, **f**, forearm radial artery (RA). The red lines, circles, arrowheads indicate the diameter, depth, cross-sectional area of the arteries and the catheter needles, respectively

In the short-axis plane, the cross-sectional area was obtained from a dotted adjustable ellipse along the vascular wall on the ultrasound image. The size of the area was calculated automatically. In the long-axis plane, the diameter and depth were measured. The diameter was considered as the distance between the anterior and posterior points of the artery wall. The subcutaneous depth was determined by the distance from the skin surface to the arterial anterior wall (Fig. 1).

**Fig. 2:**
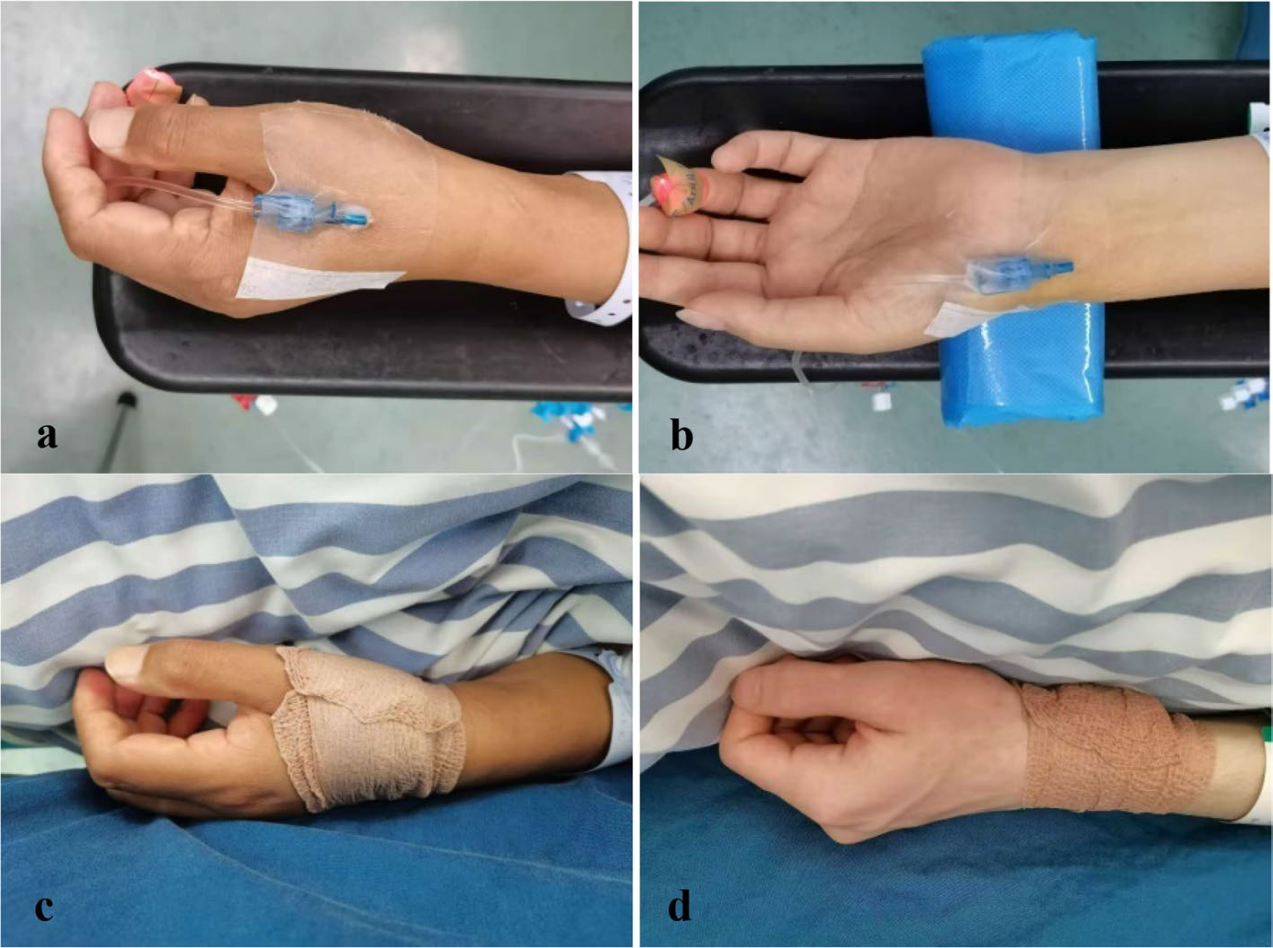
Arterial catheterization and postoperative hemostasis. **a**, **c**, distal radial artery (dRA); **b**, **d**, forearm radial artery (RA). The puncture sites located at anatomical snuffbox and the proximal 1-2 cm of the styloid process on dRA and RA, respectively

After data collection, arterial catheterization was performed by an anesthesiologist (J. X.) who was experienced in both puncture sites with real-time ultrasound-guided procedure. The area around the anatomic landmark was prepared and covered with a piece of sterile drape, and 0.5 mL of 2.0% lidocaine was administered around the puncture site. Using the long-axis in-plane ultrasound guidance, a 22-gauge catheter (B.Braun, Germany) was carefully inserted. The regions located at anatomical snuffbox and the proximal 1-2 cm of the styloid process were used as the puncture sites on dRA and RA, respectively. All catheters were connected to pressure transducers (B.Braun, Germany) flushed with heparinized saline. Pressure waveforms displayed on the patient monitors (Mindray, China) were evaluated frequently intraoperatively. The catheters were removed after the surgery. Then the puncture sites were covered by a piece of sterile gauze and rolled up 3–5 turns elastic bandages (3 M, Germany) with moderate pressure for hemostasis (Fig. 2). The primary compression time was based on the minimum time in pretest, which was 135 s in dRA group and 400 s in RA group. Success of hemostasis was defined as absence of bleeding after release of bandage. If bleeding persisted, compression was continued for an additional time. The site was the rechecked until no bleeding occurred. The total time was recorded as the postoperative compression time. The hemostasis produce was performed by an independent investigator (J. Z.), and the additional compression time was determined by clinical experience.

### Study endpoints

The primary endpoint was to demonstrate whether dRA was noninferior to RA in the first attempt success rate of arterial catheterization. The first attempt was defined as the first needle passing through a new puncture site, and successful catheterization was defined as obtaining an undamped arterial waveform on the monitor screen. The secondary endpoints included the diameter, depth and cross-sectional area of vessels; artery catheterization time (the time from routine disinfection to arterial waveform confirmation on the patient monitor); arterial posterior wall puncture rate; and postoperative compression time. Dampened arterial pressure waveforms during operation and complications (vascular, neurologic and infectious complications [14]) that occurred from the operation day to postoperative day 2 were also recorded. If catheterization was not successful within 3 min, the procedure was suspended. Rescue process was performed by a senior anesthesiologist.

### Statistical analysis

Categorical variables are displayed as frequencies and percentages. Normally distributed continuous variables are presented as mean ± SD, while non-normal distributed continuous variables are expressed as median with interquartile range. The Kolmogorov–Smirnov test was used to assess the normality of data. For primary endpoint, we used a two-sided 95% CI to test the noninferiority hypothesis. Noninferiority would be declared if the lower boundary of a 95% CI was larger than − 15%. For secondary endpoints, categorical variables were analyzed by the chi-square test or the Fisher’s exact test, and continuous variables by the independent *t* test or the Mann–Whitney *U* test. Correlations were calculated by the Pearson correlation coefficient. All statistical analyses were conducted by the SPSS 25.0 (IBM, USA) for Windows. *P* < 0.05 was considered as statistically significant.

According to statistical views, noninferiority margin should not exceed a fifth of the control rate when comparing two sample rates. In our pilot study, the initial success rate in the conventional RA approach was 90%; hence, we selected 15% as the margin after considering both statistical advice and clinically acceptable range. Utilizing this noninferiority margin with a significance level of 0.05 and power of 0.9, we computed a minimum sample size of 138 participants using PASS 15.0 (NCSS, USA). To allow a 10% dropout rate, 154 participants were needed.

## Results

**Fig. 3:**
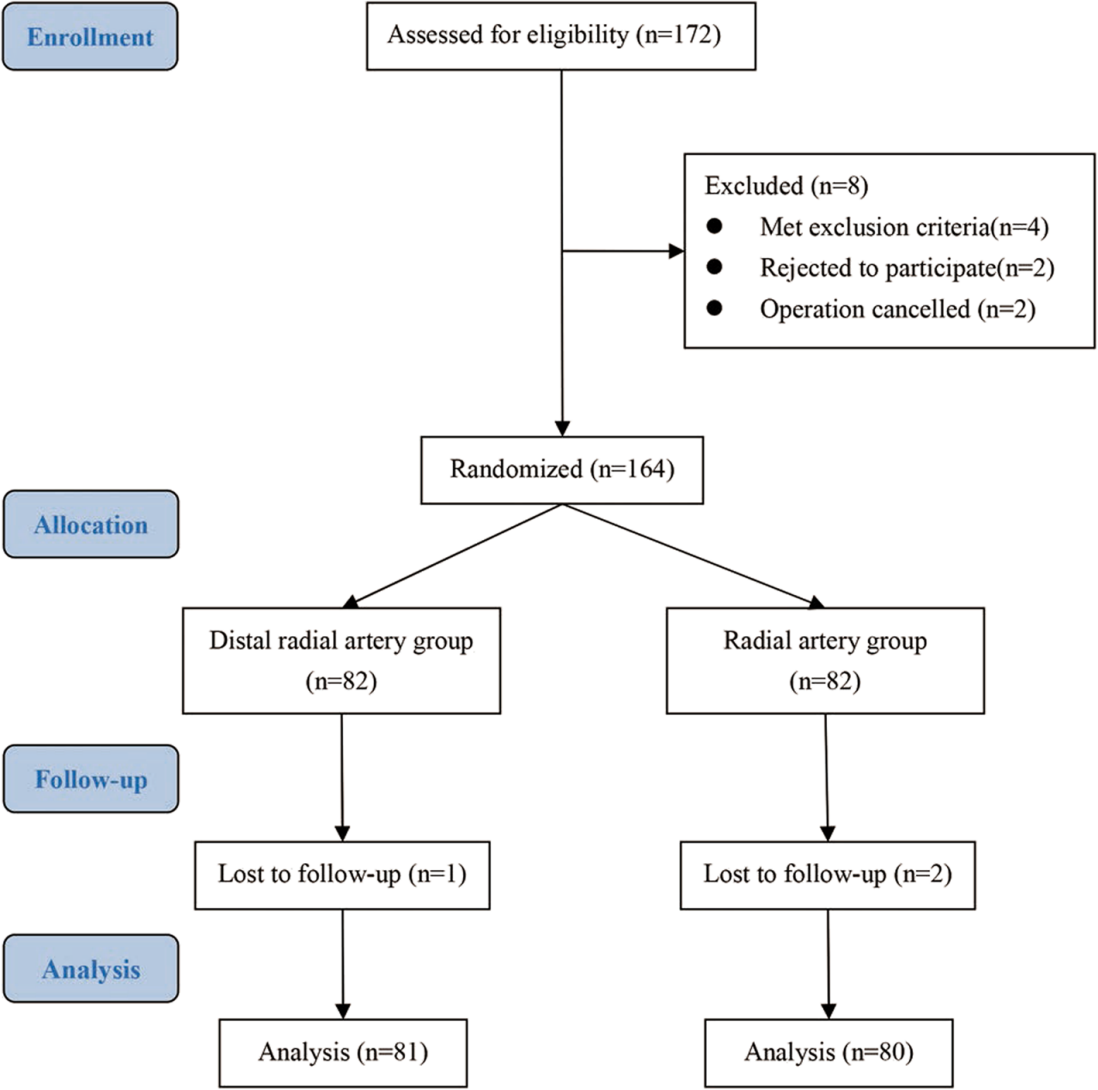
Study flowchart

We enrolled 172 patients in this study. Eight patients dropped out for the following reasons: discovery of exclusion criterion (*n* = 4); patient’s withdrawal from participation (*n* = 2); and case canceled (*n* = 2). Hence, 164 patients were randomized into the dRA and RA groups. All catheterizations were completed within 3 min. One patient in the dRA group and two in the RA group were lost to follow-up due to early discharge on postoperative day 1. In total, 161 patients were selected in the final analysis (Fig. 3). The demographic characteristics of patients between the dRA group (*n* = 81) and RA group (*n* = 80) were comparable (Table 1).

**Table 1:** Baseline characteristics

	dRA group(*n* = 81)	RA group(*n* = 80)	*P* value
Age, years	52 ± 16	51 ± 13	0.669
Male sex (%)	51(63.0)	42(52.5)	0.197
Weight, kg	66.4 ± 9.9	67.0 ± 12.4	0.687
Height, cm	166.4 ± 6.9	166.9 ± 7.1	0.884
BMI, kg/m^2^	23.9 ± 3.1	23.9 ± 3.5	0.891
Smoking status			
Never smoked, n (%)	65(80.2)	60(75.0)	0.591
Current smoker, n (%)	12(14.8)	13(16.2)	
Previous smoker, n (%)	4(4.9)	7(8.8)	
Comorbidities			
Coronary artery disease, n (%)	3(3.7)	1(1.3)	0.620
Hypertension, n (%)	14(17.3)	17(20.0)	0.523
Diabetes mellitus, n (%)	8(9.9)	4(5.0)	0.239
Arrhythmia, n (%)	1(1.2)	2(2.5)	0.620
Cerebrovascular disease, n (%)	1(1.2)	0(0.0)	1.000
CKD, n (%)	0(0.0)	1(1.3)	0.497
Medications			
Antihypertensive drugs, n (%)	15(18.5)	17(21.2)	0.664
Hypoglycemic drugs, n (%)	8(9.9)	3(3.7)	0.123
Anticoagulant or antiplatelet, n (%)	3(3.7)	2(2.5)	1.000
Others, n (%)	6(7.4)	3(3.7)	0.495
Previous artery access, n (%)	9(11.1)	11(13.8)	0.612
ASA Physical Status Class			
I, n (%)	8(9.9)	5(6.3)	0.781
II, n (%)	72(88.9)	74(92.5)	
III, n (%)	1(1.2)	1(1.3)	
Operation position			
Supine, n (%)	41(50.6)	44(55.0)	0.090
Lateral, n (%)	17(21.0)	25(31.3)	
Prone, n (%)	9(11.1)	6(7.5)	
Lithotomy, n (%)	14(17.3)	5(6.3)	
Operation time, min	120(81)	115(83)	0.999

*BMI* body mass index, *ASA* American Society of Anesthesiologists, *CKD* chronic kidney disease. Data are displayed as the mean ± SD, frequency and percentage, median (interquartile range)

**Fig. 4:**
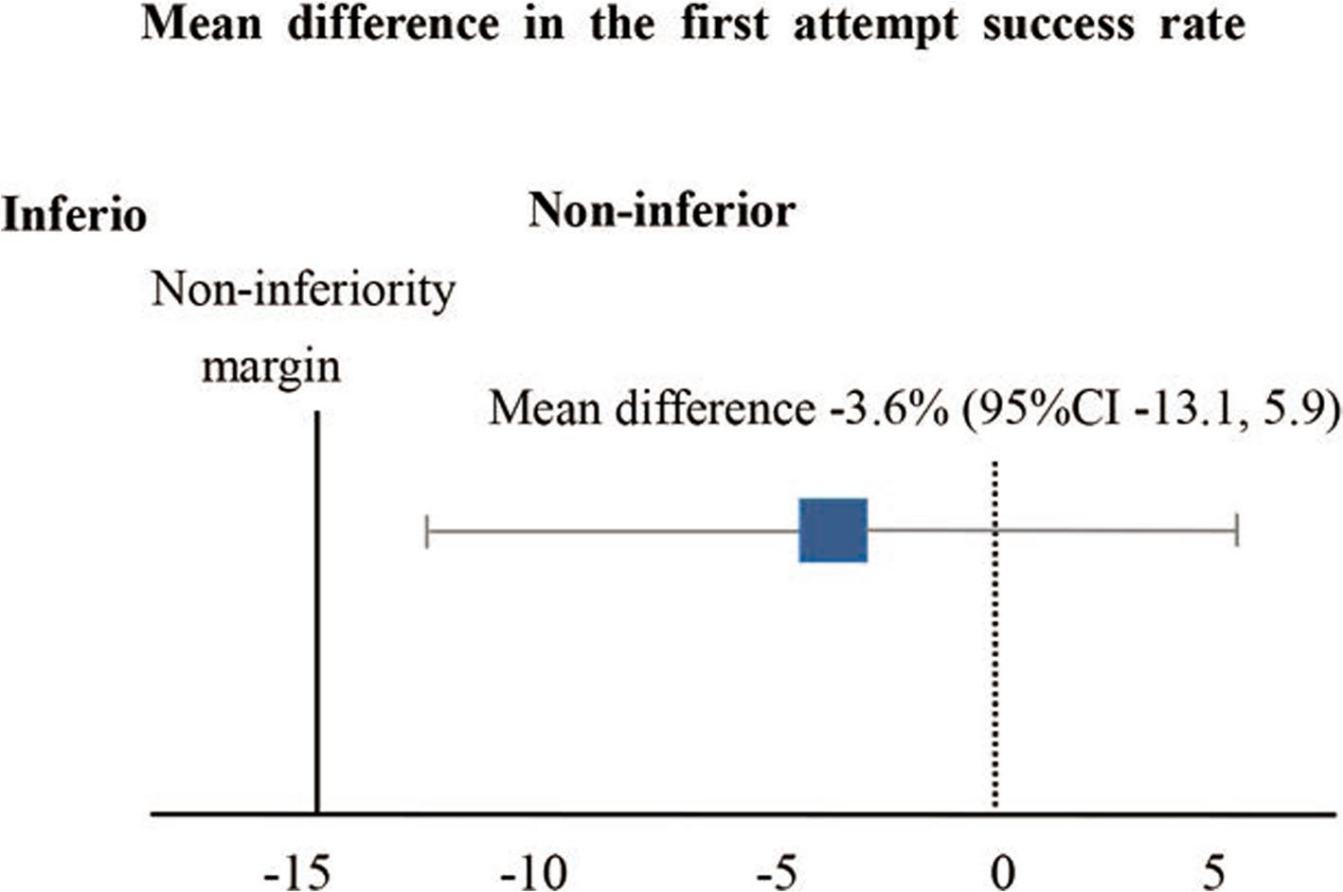
Mean difference in first attempt success rate. CI, confidence interval. Black vertical continuous line indicates noninferiority margin. The right part of the black line indicates noninferiority

The first attempt success rates were 87.7 and 91.3% in the dRA and RA groups, respectively, with a mean difference of − 3.6% (95% CI, − 13.1 to 5.9%). Since the noninferiority margin was defined as − 15%, the dRA group proved a noninferior first attempt success rate to the RA group (Fig. 4).

Table 2 shows the specific ultrasound measurement results. The dRA diameter and cross-sectional area were both significantly smaller than those of the RA (*P* < 0.001). Meanwhile, the subcutaneous depth of the dRA was significantly deeper than that of the RA (*P* < 0.001). The correlation indexes between the diameter and body weight in the dRA and RA groups were significantly positive (*r* values were 0.465 and 0.401, respectively; *P* < 0.001). However, the correlation between the diameter of the dRA and RA was relatively poor (*r* = 0.053, *P* = 0.641).

**Table 2:** Ultrasound examination results

	dRA group(*n* = 81)	RA group(*n* = 80)	*P* value
Diameter, mm	2.1 ± 0.4	2.6 ± 0.4	< 0.001
Depth, mm	2.5 ± 0.5	1.8 ± 0.4	< 0.001
Cross-sectional area, mm ^2^	3.9 ± 1.0	5.6 ± 1.6	< 0.001

The measuring point of the dRA was at the anatomical snuffbox; RA in the proximal 1-2 cm of the styloid process of the radius. Data are displayed as the mean ± SD

Procedural data are summarized in Table 3. The catheterization was performed from the left arm in 106 patients, without differences between both groups (*P* = 0.824). The time to catheterization was statistically longer in the dRA group (*P* = 0.008). Nevertheless the postoperative compression time was significantly shorter in the dRA group than in the RA group (*P* < 0.001). The arterial posterior wall puncture rate of the dRA was significantly higher than that of the RA (21% vs 6.3%, *P* = 0.006). The dRA had fewer intraoperative dampened arterial waveforms than RA (*P* = 0.030). The complications did not differ between the two groups until postoperative day 2 (*P* = 0.497). There was one patient who presented with a 2 × 1 cm^2^ local hematoma in the dRA group after catheter removal. In the RA group, one patient had puncture site discoloration and one patient had vasospasm. No neurological complications or puncture site infections were found in either group.

**Table 3:** Procedural data

	dRA group(*n* = 81)	RA group(*n* = 80)	*P* value
Left hand, n (%)	54(66.7)	52(65)	0.824
First attempt success rate, n (%)	71(87.7)	73(91.3)	0.458
Arterial catheterization time, s	86(26)	74(25)	0.008
Postoperative compression time, s	199 ± 44	486 ± 56	< 0.001
Arterial posterior wall puncture, n (%)	17(21.0)	5(6.3)	0.006
Dampened arterial waveforms, n(%)	8(9.9)	18(22.5)	0.030
Complications			
Discoloration, n (%)	0(0)	1(1.3)	0.497
Vasospasm, n (%)	0(0)	1(1.3)	
Hematoma, n (%)	1(1.2)	0(0)	

Data are displayed as the mean ± SD, frequency and percentage, median (interquartile range)

## Discussion

Forearm radial arterial catheterization is the classic access for invasive hemodynamic monitoring. Common alternative approaches include the femoral artery, brachial artery, ulnar artery, and dorsalis pedis artery. However, all approaches have their own limitations. The femoral artery has disadvantages of difficultly of compression and major complications [14, 15]. The brachial artery lacks collateral flow, thereby making it an ill fit to be the site of an indwelling arterial cannula [16]. The ulnar arterial puncture has a deeper location, leading to risk of ulnar nerve injury [17]. The dorsalis pedis artery, which is always chosen in the lower extremity, has an obvious blood pressure gradient in comparison with the radial artery [18]. Thus, dRA has attracted our attention.

DRA crosses relatively superficially at the anatomical snuffbox and receives collateral circulation from the superficial palmar branch and the ulnar artery [9]. The dRA access is considered to maintain the integrity of the forearm radial artery and conserve it for future interventions [19].

Our study first reported a comparison of dRA and RA catheterization for invasive blood pressure monitoring. The major finding in the current study was that dRA offered a noninferior initial success rate to RA in the general population. Kaledin et al. reported that 12.5% of dRA catheterizations required more than one attempt [20]. Arora et al. showed that the rate of first-pass radial artery was 85.7% via the in-plane technique in awake patients [21]. In the present study, the initial success rates of dRA and RA were 87.7 and 91.3%, respectively. Furthermore, both groups had 100% final success rates within 3 min. This is consistent with the results of previous meta-analyses [22, 23].

Measuring the anatomical characteristics of the vascular area by ultrasound is an effective, economic, and optimal method [24]. Anatomically, the dRA is slightly smaller and deeper than the RA, and catheterization can be challenging. Although a learning curve is needed, ultrasonic guidance may help us overcome this weakness and facilitate the puncture of small vessels. Despite a time-consuming catheterization, the compression time of dRA is remarkably shorter, which is possibly because of the smaller size and easier compression [25]. More recent studies have supported these results [26, 27].

Another important finding in this trial was that the dRA had fewer arterial pressure waveform abnormalities than RA after changing the position. Nearly half of operation position was not supine in this study. Especially in the prone position, the pronated palm faces the arm board, and minor changes in wrist angle can damp the arterial waveform in RA. However, in dRA, the arterial waveform is less affected by malposition because it is firmly supported by the scaphoid and trapezium bones, and an inserted catheter will stand at a stable position [7, 28].

In some complex radial cases, patients with elbow joint diseases, contractures, trauma or any congenital abnormalities, who keep the arm supinated for a long time, may experience discomfort [26]. However, dRA access may provide a more comfortable position for a pronated arm.

Furthermore, considering the vessel compensation system of the hand, dRA catheterization could have a lower risk of ischemia than RA. In fact, the dRA approach also has other complications, including hematoma and nerve injury [23]. A higher occurrence of complications is often related to multiple punctures [29]. The complications between the two study groups in our trial did not significantly differ probably because of our high success rate on the first try. To prove this, larger samples and multicenter experiences are needed.

This study had some limitations. First, our results were obtained from one single center, and it should be taken into consideration when trying to promote these findings to other clinical institutions. Second, the puncturing investigator and patients were not blinded to the performance, which could potentially lead to observer variability. Third, only one investigator completed all arterial catheterizations, and this could lead to difficulty of generalizing the results. However, our puncturing investigator had anesthetic experience of > 5 years and had performed arterial catheterization in more than 100 cases with ultrasound guidance [10, 30]. This level of experience is common in the majority of anesthesiologists worldwide.

## Conclusions

In conclusion, the distal radial artery can be a rational choice for perioperative arterial blood pressure monitoring. However, further evaluation is needed to evaluate its potential to replace the forearm radial artery as the default approach.

## Data Availability

All data generated or analysed during this study are included in this published article.

## Abbreviations

dRA: Distal radial artery
RA: Radial artery
ASA: American Society of Anesthesiologists
BMI: Body mass index
ECG: Electrocardiograph
HR: Heart rate
SpO_2_: Oxygen saturation
